# Survey of the bacteriophage *phoH* gene in wetland sediments in northeast China

**DOI:** 10.1038/s41598-018-37508-4

**Published:** 2019-01-29

**Authors:** Xiang Li, Yan Sun, Junjie Liu, Qin Yao, Guanghua Wang

**Affiliations:** 1 Key Laboratory of Mollisols Agroecology, Northeast Institute of Geography and Agroecology, Chinese Academy of Sciences, Harbin, 150081 China; 2 University of Chinese Academy of Sciences, Beijing, 100049 China

## Abstract

*PhoH* is a host-derived auxiliary metabolic gene that can be used as a new biomarker for surveying phage diversity in marine and paddy waters. However, the applicability of this gene in other environments has not been addressed. In this paper, we surveyed the *phoH* gene in four wetland sediments in northeast China. DNA was extracted directly from sediments and used for PCR amplification with the degenerate primers vPhoHf and vPhoHr. In total, 44 and 58 *phoH* sequences were identified as belonging to bacteria and phages, respectively, suggesting that this primer set is not highly specific to the phage *phoH* gene. A BLASTp search showed that the 58 phage *phoH* sequences had the highest identity to the known viral sequences, ranging from 48% to 100%. Phylogenetic analysis showed that all phage sequences from wetlands distributed into the previously designated Groups 2, 3, 4 and 6. In addition, two new subgroups, Groups 2c and 4c, which contained sequences exclusively from wetlands, were detected in this study. Nonmetric multidimensional scaling analysis showed that the phage *phoH* assemblage from a coastal wetland was similar to that in marine environments, while the phage *phoH* assemblage from a lake wetland was similar to that in paddy waters. These findings indicated that different types of wetlands had distinct phage *phoH* compositions.

## Introduction

Phage auxiliary metabolic genes (AMGs) were originally from the genomes of cellular microorganisms but have been detected in several phage genomic sequences^1^. The sequences of phage AMGs are often homologous to their host genes, but phylogenetic analysis can separate AMGs from hosts^2^. Therefore, several AMGs can be used as biomarker genes to study phage diversity in natural environments^3^. These AMGs include the photosynthetic genes *psbA* and *psbD*, which encode the photosystem D1 and D2 proteins^4–6^; the *mazG* gene, which encodes pyrophosphatase^7^; and the *phoH* gene, which is relates to phosphate metabolism^8^.

*PhoH* has some advantages as a biomarker gene over other biomarker genes for studying phage diversity, such as *g23*^9^, *g20*^10^, DNA *pol*^11^ and *psbA*^4^, since those genes are restricted to specific morphological phages. The *phoH* gene was detected in various morphological types of phages (including *siphophages*, *myophages* and *podophages*) and in a wide host range (including autotrophic and heterotrophic bacteria) and even in the viruses of autotrophic eukaryotes^8^. By using this gene, viral *phoH* sequences in the Sargasso Sea and worldwide oceans were clustered into six novel groups (Groups 1~6). In addition, the distribution patterns of viral *phoH* assemblages in the Sargasso Sea differed with water depth and sampling time^12,13^. Although the *phoH* gene was detected in 40% of cultured marine phages and in only 4% of cultured nonmarine phages^8^, it appears that this gene is not suitable for assessing the diversity of phages in terrestrial environments. However, more than 400 phage *phoH* sequences were recently obtained from several paddy floodwaters in northeast China, and 4 specific groups and 7 subgroups of phage *phoH* were detected in paddy waters^14^. These findings suggest that this biomarker gene can be used to investigate the diversity of phages in both marine and terrestrial environments.

Wetlands are ecotones between uplands and water bodies characterized by shallow water that is permanently or periodically close to the soil surface, and they establish specific wetland plant communities^15,16^. Wetlands have a functional role in several key biogeochemical processes, such as pollutant degradation, nitrification, denitrification, methanogenesis, methanotrophy, and iron and sulfate reduction^17,18^. Thus, wetlands are usually considered the “kidney” of the earth. Since these biogeochemical processes are mostly mediated by microbes^19^, wetlands are regarded as the hotspots for studying microbial ecology^18^; however, assessments of virus or phage diversity in wetlands are limited^20,21^. In this study, by targeting the biomarker gene *phoH*, we surveyed phage diversity and distribution in several wetland sediments in northeast China using culture-independent PCR, cloning and Sanger sequencing methods. The objectives were (i) to test whether the phage *phoH* gene can be obtained from natural wetlands; (ii) to compare the phage *phoH* sequence diversity and novelty with known sequences; and (iii) to compare the difference or similarity of phage *phoH* assemblages in natural wetlands with that in marine and paddy fields.

## Results

### PCR amplification and the closest relatives of *phoH* clones

Using degenerate primers vPhoHf and vPhoHr, PCR products were generated from wetland sediments obtained from two coastal wetlands of Liaohekou (LHK) and Yalujiangkou (YLJK), a lake wetland of Xingkaihu (XKH), and a swamp wetland of Honghe (HH), respectively. Electrophoresis revealed several faint bands of PCR products were showed on the gel (Supplementary Fig. S1). Only bands with a target length of approximately 420 bp were excised, purified and cloned for sequencing.

In total, 219 positive clones were submitted for sequencing, but only 102 clones were confirmed as *phoH* sequences. A BLASTp search at the amino acid level indicated that 44 clones had the highest identities ranging from 78% to 96% to bacterial strains and environmental bacterial clones, with exception of the clone LHK-phoH-7 and clone HH-phoH-3, which had 51% identity to *Spirochaetes* bacterium GWB1_48_6^22^ and *Methylobacterium extorquens* AM1^23^, respectively (Supplementary Table S1). The remaining 58 clones had the highest identities ranging from 48% to 100% to environmental phage clones obtained from paddy water^14^ and sea waters^8^, as well as marine cultured *Synechococcus* phages^24–26^ (Supplementary Table S2).

### Phylogenetic analysis of *phoH* sequences

**Figure 1:**
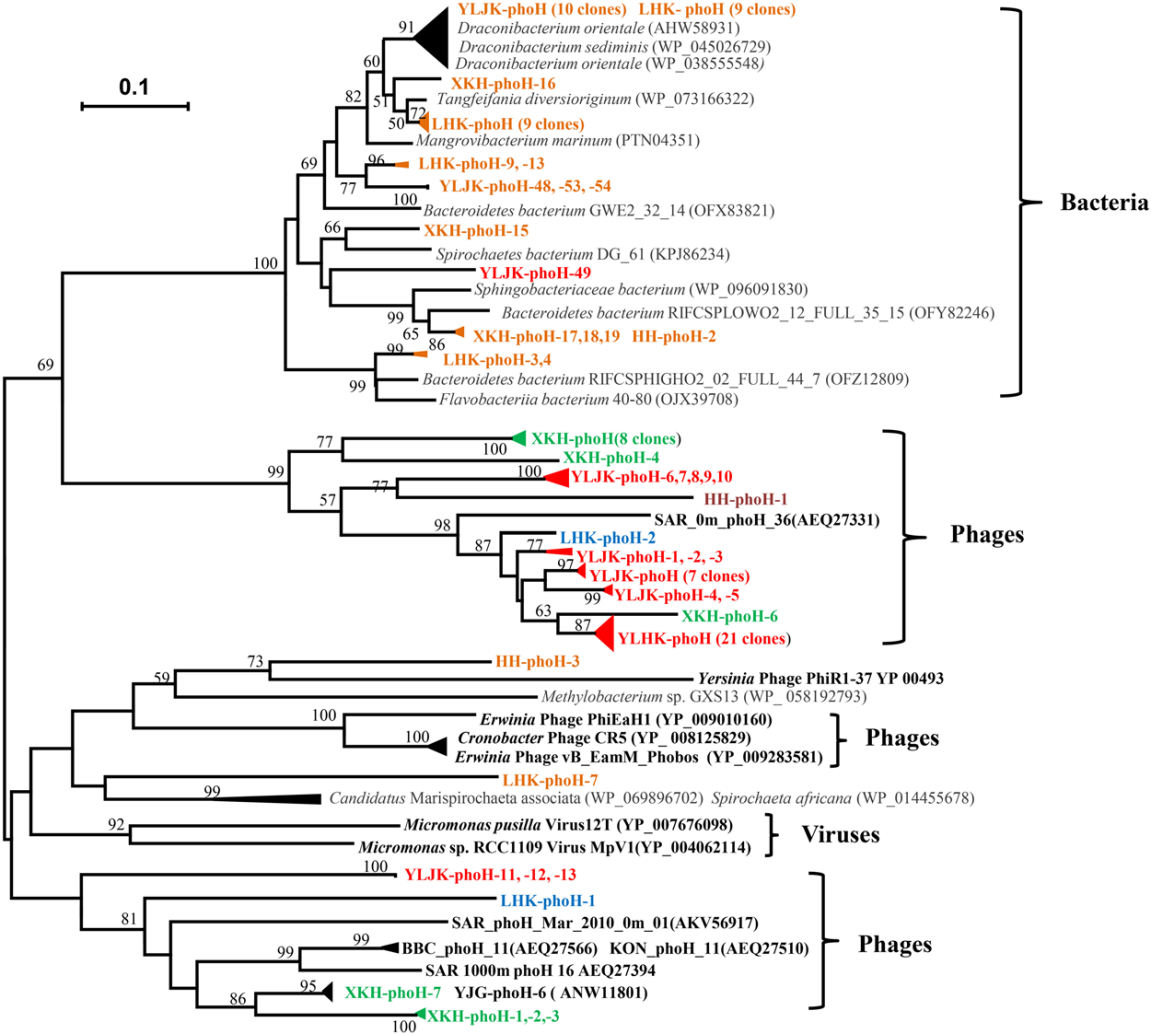
Neighbor-joining phylogenetic tree constructed with *phoH* sequences obtained in wetland sediments and their closest relatives retrieved from GenBank at the amino acid level. Numbers in parentheses are the accession numbers of *phoH* sequences in the NCBI website. The *phoH* sequences from bacteria and viruses (phages) are shown in normal gray letters and bold black letters, respectively. The bootstrap support values < 50 are not shown. The scale bar represents a 10% difference in amino acid sequence.

A neighbor-joining phylogenetic tree was constructed from the *phoH* sequences isolated in this study and the *phoH* sequences from the closest relatives identified in Blastp (Fig. 1). The tree showed that all clones in Supplementary Table S1 formed a large cluster with a 100% bootstrap support value with bacterial *phoH* sequences, with the exception of two clones, HH-phoH-3 and LHK-phoH-7. Thus, these clone sequences were considered *phoH* sequences obtained from wetland bacteria. The clones in Supplementary Table S2 formed two clusters with bootstrap support values of 99% and 48% (data not shown in the Figure), respectively, with environmental viral clones or phages; therefore, those clones were thought to be obtained from wetland viruses. The two clones HH-phoH-3 and LHK-phoH-7 had 51% identity to the *phoH* sequences of bacteria (Supplementary Table S1) but formed several low bootstrap-supported branches with *phoH* sequences of bacteria and phages. Thus, the origin of clones HH-phoH-3 and LHK-phoH-7 could not be determined.

**Figure 2:**
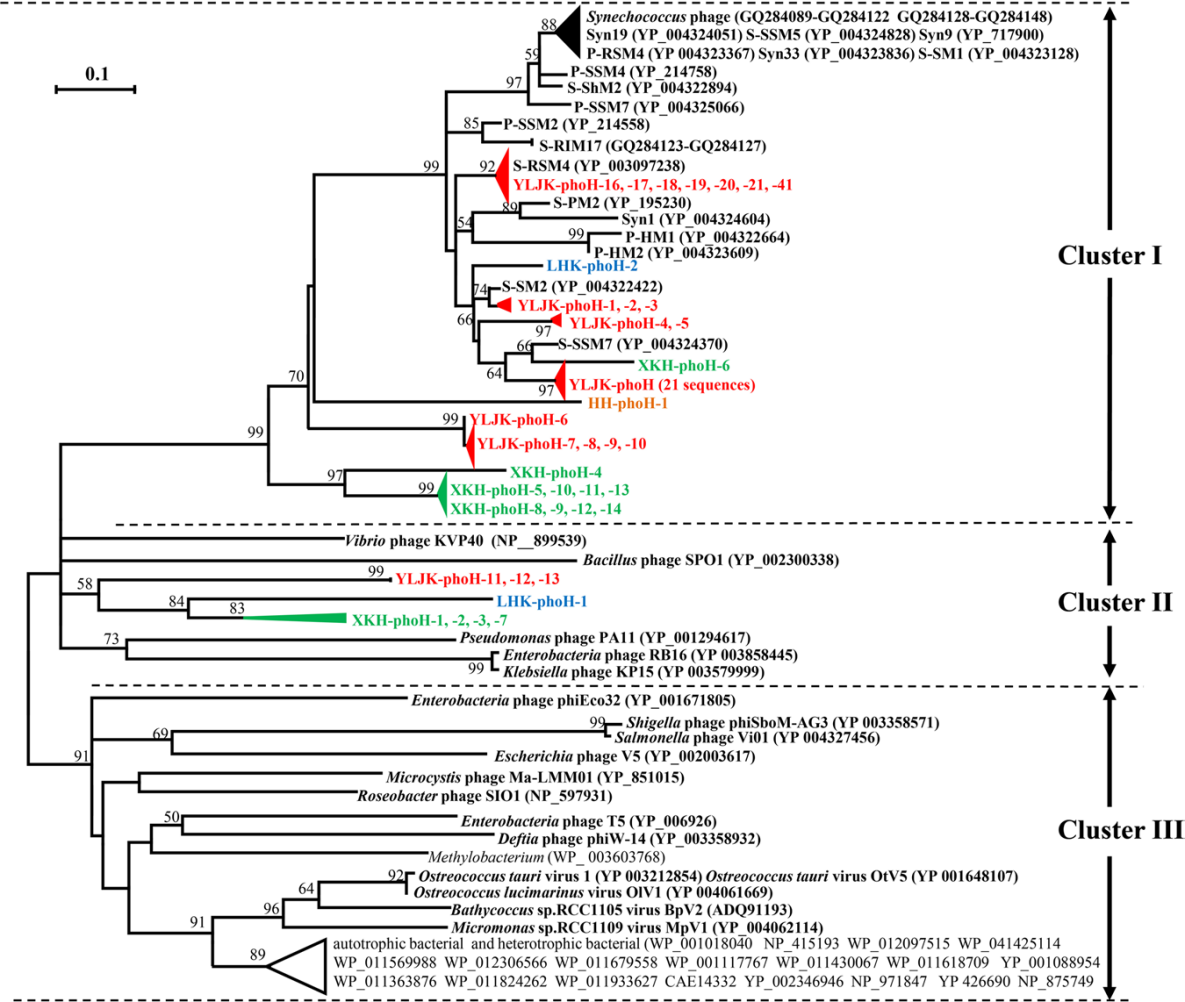
Neighbor-joining phylogenetic tree constructed with phage *phoH* sequences obtained in wetland sediments and the *phoH* sequences of cultured bacteria, phages and eukaryotic viruses at the amino acid level. The *phoH* sequences from cultured phages of *Synechococcus*, *Prochlorococcus* and heterotrophic bacteria are shown in bold black letters, while the *phoH* sequences from cultured bacteria are shown in normal black letters. Numbers in parentheses are the accession numbers of *phoH* sequences in the NCBI website. Bootstrap support values < 50 are not shown. The scale bar represents a 10% difference in amino acid sequence.

Through the above exclusion analysis, 58 viral *phoH* sequences were obtained from wetland sediments. Among them, 41, 2, 1 and 14 sequences were from sediments of YLJK, LHK, HH and XKH, respectively. The identity among 58 viral phoH sequences at amino acid level ranged from 22% to 99%, which indicates that the diversity of viral phoH gene in wetland sediments is high. To determine the phylogenetic position of viral *phoH* sequences in wetlands, 58 viral *phoH* sequences obtained in this study were used to build a phylogenetic tree with *phoH* sequences from cultured phages of *Synechococcus*, *Prochlorococcus* and heterotrophic bacteria, as well as the *phoH* sequences from autotrophic and heterotrophic bacteria (Fig. 2). All sequences were divided into three clusters. Cluster I contained 50 clones obtained in this study with phages of autotrophic bacteria, and Cluster II included 8 clones obtained in this study with phages of heterotrophic bacteria. None of the phage *phoH* sequences in this study fell into Cluster III. Cluster III comprised the viruses infecting eukaryotes, phages infecting several heterotrophic bacteria, a phage Ma-LMM01 of *Microcystis*, and some bacterial *phoH* sequences. Thus, all 58 viral *phoH* sequences obtained in this study were considered to come from phages.

**Figure 3:**
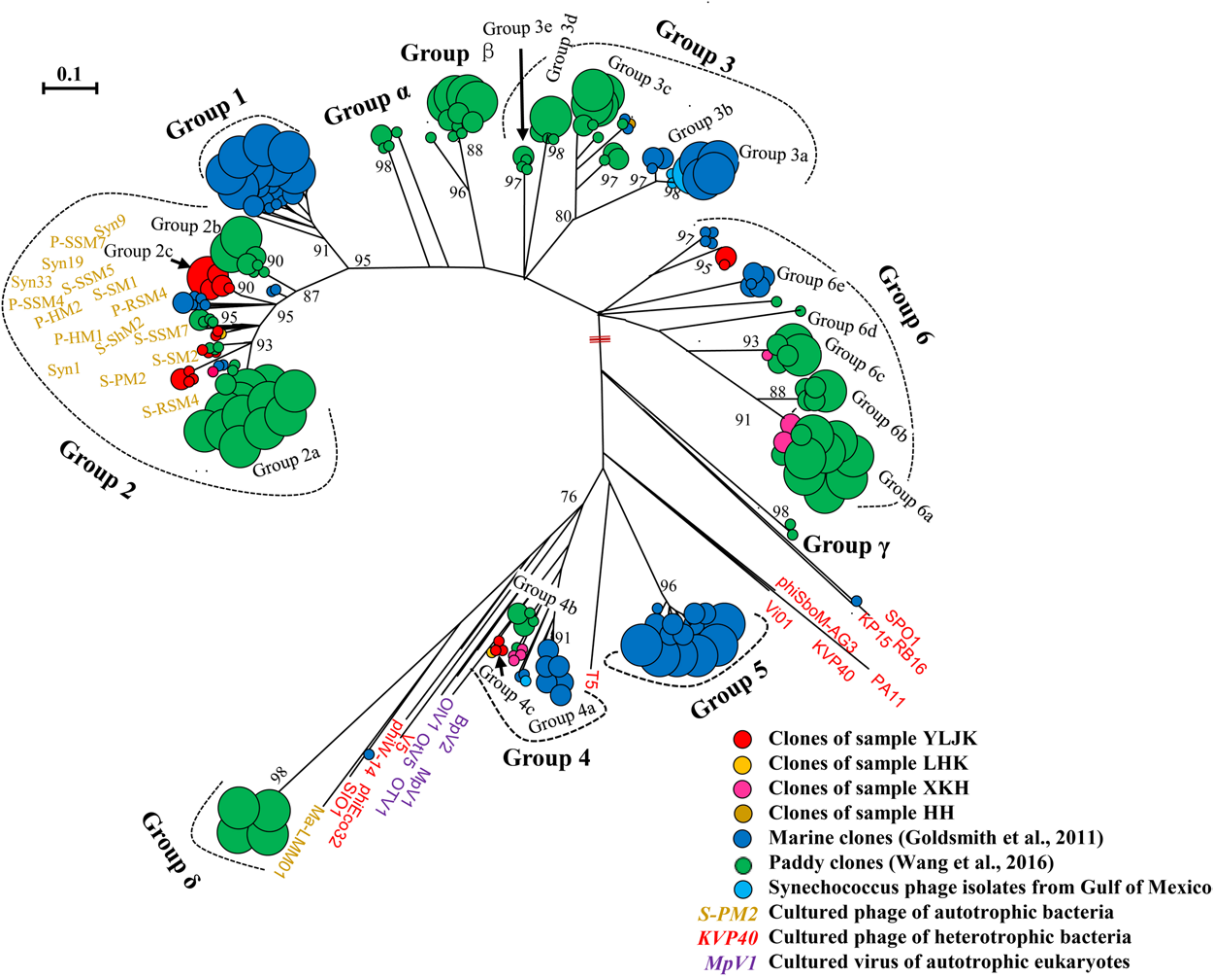
Unrooted phylogenetic tree showed the relationships of phage *phoH* amino acid sequences obtained from environmental clones of wetlands in this study, marine waters (Goldsmith *et al*. 2011) and paddy waters (Wang *et al*. 2016), and cultured phages and cultured eukaryotic viruses. The size of the circles at the end of the branches is proportional to the number of clones/phages, and the small, medium and large circles represent one, four and eight clones/phages, respectively. The bootstrap support values < 50 are not shown. The scale bar represents a 10% difference in amino acid sequence.

To test whether novel groups of phage *phoH* sequences exist in wetlands, a phylogenetic tree at the amino acid level was constructed with the phage *phoH* sequences obtained in this study, marine waters^8^, paddy waters^14^, cultured phages of autotrophic and heterotrophic bacteria, and cultured viruses of autotrophic eukaryotes (Fig. 3). Based on the grouping standard reported previously^8,14^, 35, 1, 8 and 14 clones from wetlands fell into Groups 2, 3, 4 and 6, respectively. Within Groups 2 and 4, two new subgroups, Group 2c and Group 4c, which contained 21 and 4 clones exclusive to this study, were identified. The proportional distributions of phage *phoH* clones across different groups in wetland sediments, marine waters and paddy waters are summarized in Table 1, which indicates that the distribution of this biomarker gene differed among the three environments.

**Table 1:** Number and proportional distribution of phage *phoH* clones in phylogenetic groups obtained from marine waters, paddy waters and wetland soils.

Phylogenetic groups	Marine^a^ (275)^d^		Paddy^b^ (424)^d^		Wetland^c^ (58)^d^	
	Number of clones	Proportion (%)	Number of clones	Proportion (%)	Number of clones	Proportion (%)
Group α			6	1.42		
Group β			49	11.56		
Group γ			2	0.47		
Group δ			32	7.55		
Group 1	91	33.09				
Group 2a	13	4.73	102	24.05	14	24.14
Group 2b			27	6.37		
**Group 2c** ^e^					21	36.21
Group 3a	33	12				
Group 3b	8	2.91				
Group 3c	2	0.73	48	11.32	1	1.72
Group 3d			14	3.30		
Group 3e			7	1.65		
Group 4a	4	1.45				
Group 4b	24	8.72	11	2.59	4	6.90
**Group 4c** ^e^					4	6.90
Group 5	77	28				
Group 6a			72	16.98	8	13.79
Group 6b			24	5.66		
Group 6c			28	6.60	1	1.72
Group 6d			1	0.24		
Group 6e	21	7.64	1	0.24	5	8.62
Ungrouped	2	0.73				

^a^Marine sources, including the Sargasso Sea and worldwide oceans (Goldsmith *et al*., 2001).

^b^Paddy sources, including the four paddy sampling sites obtained from northeast China (Wang *et al*., 2016).

^c^Wetland source of this study.

^d^Number in parenthesis is the total number of clones obtained from each source.

^e^Group in bold is a newly designated subgroup in this study.

### Biogeography of phage *phoH* sequence assemblage

**Figure 4:**
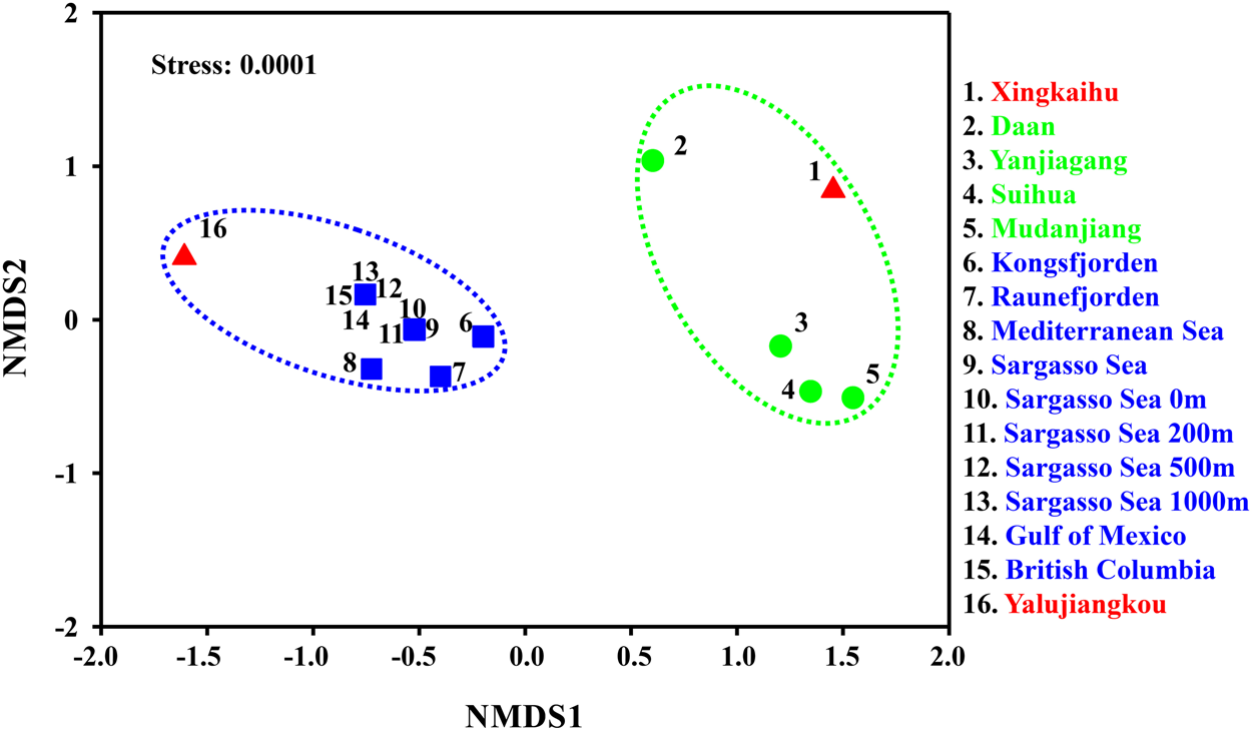
A nonmetric multidimensional scaling plot showing the distribution pattern of the phage *phoH* assemblages obtained from different environments. Samples located close to each other on the plot are grouped by dashed circles.

To characterize the relationships of phage communities in different environments, the phage *phoH* sequence assemblages from two wetland sediments of this study, 10 marine waters reported previously^8^, and four paddy waters of northeast China^14^ were evaluated with nonmetric multidimensional scaling (NMDS) analysis (Supplementary Table S3). Of note, since only two phage *phoH* clones from LHK and one clone from HH were obtained in this study, these two wetland samples were not used for NMDS analysis. Overall, the *phoH* assemblages from different environments can be divided into two major groups: samples from YLJK and marine waters fell into one group, while samples from XKH and paddy waters fell into the other group (Fig. 4).

## Discussion

The degenerate primers vPhoHf and vPhoHr were first designed based on alignment of the full-length *phoH* gene from *Synechococcus* phage S-PM2, *Prochlorococcus* phages P-SSM2 and P-SSM4, as well as *Vibrio* phage KVP40^8^. Using this primer set, Goldsmith *et al*.^8,12^ successfully obtained many phage *phoH* sequences from various marine waters. Following that, our research group further isolated 424 sequences of phage *phoH* from four paddy floodwaters in northeast China, indicating that this primer set was applicable for studying phage diversity in both marine and paddy waters^14^. It should be noted that the water samples used for the above studies excluded host DNA using the ultracentrifuge or Millipore filtering method; we do not know whether this primer set can be applied to studies of nonwater environments. In this study, we extracted microbial DNA directly from wetland sediments as previously conducted for studying the diversity of the *g23* gene of T4-type phage^27^. The extracted DNAs were used as a template for PCR amplification, and the results showed that 44 sequences had the highest identity to bacterial strains (Supplementary Table S1). Phylogenetic analysis also separated these sequences from phage or virus groups (Fig. 1), suggesting that these sequences originated from bacteria. This finding conflicted with Goldsmith *et al*.^12^, whom stated that this primer set does not amplify known bacterial *phoH* genes. To the best of our knowledge, this is the first report to reveal that host *phoH* genes can also be amplified with the primers vPhoHf and vPhoHr. This finding underscores the importance of excluding host *phoH* sequences when investigating viral or phage *phoH* genes in natural environments.

It should be noted that both clones LHK-phoH-7 and HH-phoH-3 had 51% identity to *phoH* sequences of known bacterial strains (Supplementary Table S1), and HH-phoH-3 had 53% identity to *Yersinia* phage phiR1-37 (YP_004934283) (Supplementary Table S2); however, both clones fell into two branches with bacteria, although the bootstrap supporting value was lower than 50% (Fig. 1). Based on this finding, the origins of clones LHK-phoH-7 and HH-phoH-3 remained unclear. The fact that 58 clones of phage *phoH* genes were obtained in wetland sediments after excluding potential host *phoH* sequences indicated that this primer set can be used to assess phage ecology in soil ecosystems.

Although AMGs of phages were derived from their host counterparts, many of these genes, such as *psbA*^28^, *psbD*^29^ and *mazG*^7^, are evolving differently from their hosts, resulting in phages and hosts residing in different phylogenetic branches or clusters. Likewise, Goldsmith *et al*.^8^ demonstrated that *phoH* sequences coming from phages could be separated from those coming from hosts by constructing a phylogenetic tree, and phages of cyanobacteria segregated into different evolutionary branches than phages of heterotrophic bacteria or viruses of eukaryotes. In this study, we found that the majority (86%) of the phage *phoH* sequences from wetlands formed a high bootstrap supporting (99%) group of Cluster I with several cyanophages of *Synechococcus* and *Prochlorococcus*. None of the *phoH* sequences from heterotrophic bacterial phages, eukaryotic viruses, or host-derived *phoH* gene sequences fell into Cluster I (Fig. 2). Thus, we deduce that the *phoH* sequences from this study in Cluster I are likely derived from phages of autotrophic bacteria, *i*.*e*., the phage of cyanobacteria. The remaining 14% of wetland phage *phoH* clones fell into Cluster II along with several *phoH* sequences of the heterotrophic bacterial phages; thus, the origins of these *phoH* sequences were considered as coming from phages of heterotrophic bacteria. Similar to our previous finding^14^, the number of clones belonging to cyanophages containing *phoH* genes was higher than that of heterotrophic bacterial phages in wetlands; thus, the AMG of *phoH* was suspected to be mainly carried by cyanophages rather than heterotrophic bacterial phages.

The phage *phoH* sequences from marine waters were clustered into six groups (Groups 1–6)^8^; however, only Group 2 contained reference *phoH* sequences of cyanophages infecting *Synechococcus* and *Prochlorococcus*^8^. Subsequently, our research group surveyed the genetic diversity of phage *phoH* in paddy waters in northeast China, and we found that the sequences from paddy waters fell into Groups 2, 3, 4, 6 and four newly designed groups (Groups α, β, γ and δ). In addition, 7 novel subgroups (Groups 2b, 3d, 3e, 6a, 6b, 6c and 6d) were established within individual groups. In this study, 58 phage *phoH* clones were obtained from the four wetland sediments, although the number was not comparable between samples. All sequences were clustered into Groups 2, 3, 4 and 6, and two new subgroups were detected within Groups 2 and 4 (Group 2c and Group 4c) (Fig. 3). None of the phage *phoH* sequences from wetland sediments were distributed into Groups α~δ and Group 5 (Table 1), which suggested that the distribution of phage *phoH* genes in wetlands differed from those in marine waters^8^ and paddy waters^14^.

The compositions of phage *phoH* sequences were determined to be different among six worldwide oceans^8^, among four paddy waters and between ocean and paddy environments^14^. In this study, we further revealed that the composition of phage *phoH* sequences in wetland sediments was distinctly different from that in marine waters and paddy waters (Table 1). This finding to some extent was in accord with the results of another study using the biomarker gene *g23* of T4-type phages in wetlands^20^, which suggested that the distribution of phage biomarker genes in wetlands differed from that in other environments.

Our previous study revealed that the phage *phoH* sequence assemblages were grouped into marine water group and paddy water group, irrespective of where those samples were obtained^14^. These two groups were also confirmed in this study, and the two wetland samples were distributed into the two groups separately, that is the samples from YLJK and XKH fell into the marine water group and paddy water group, respectively (Fig. 4). This finding suggested that the phage communities evaluated by *phoH* sequence assemblage in coastal wetland of YLJK were similar to those in oceans, while in lake wetland of XKH, the phage communities were similar to those in paddy waters. This finding was somewhat unsurprising because the coastal wetland of YLJK was frequently influenced by tidal water of the Bohai Sea, and the lake wetland of XKH was surrounded by paddy fields. This finding supported the common opinion that “everything is everywhere, but the environment selects”^30,31^, which suggests that the selection effect is not only reflected in the bacterial community but also reflected in AMGs of phage genomes^8^.

In conclusion, this paper first demonstrated that the primers vPhoHf and vPhoHr were not highly specific to phage *phoH* genes. Through the exclusion analysis, 58 phage *phoH* clones were obtained from wetland sediments. We found that all clones were clustered into formerly known Groups 2, 3, 4 and 6. The finding of two new subclusters (Group 2c and Group 4c) containing clones exclusively obtained from wetlands in this study suggested that novel subgroups of phages inhabit wetlands. In addition, the finding that phage *phoH* assemblages of YLJK and XKH were similar to those in marine waters and paddy waters, respectively, suggested that the distribution patterns of phage *phoH* sequences were distinctly different between coastal wetland and lake wetland.

## Materials and Methods

### Wetland sediment sampling

Four wetland sediment samples, including two coastal wetlands of Liaohekou (LHK) and Yalujiangkou (YLJK), a lake wetland of Xingkaihu (XKH) and a swamp wetland of Honghe (HH) were collected across northeast China during July and August 2016. Briefly, approximately 2 kg of sediments within a soil depth of 0–10 cm were randomly collected from 5 sites in each wetland; the mixed samples were deposited into a polyethylene bag and transported to the laboratory at 4 °C. Then, some samples were loaded into 50 mL centrifuge tubes and stored at −80 °C for DNA extraction. The remaining sediment samples were air-dried for determination of physicochemical properties. The locations of sampling sites are shown in Supplementary Fig. S2 and some soil physiochemical properties of sediments are shown in Table 2.

**Table 2:** Locations of wetlands and some sediment soil properties.

Sample	Location	Latitude and longitude	Wetland type	pH	Total C (g kg^−1^)	Total N (g kg^−1^)	Total P (g kg^−1^)	Total K (g kg^−1^)	NH_4_^+^-N (mg kg^−1^)	NO_3_^–^N (mg kg^−1^)	Available P (mg kg^−1^)	Available K (mg kg^−1^)
LHK	Liaohekou, Liaoning	40°56′52.26″N, 121°47′34.98″E	Coastal	8.27	9.89	0.93	0.53	23.01	5.24	0.94	21.75	648.77
YLJK	Yalujiangkou, Liaoning	39°49′22.50″N, 124°03′32.64″E	Coastal	7.49	10.44	1.05	0.72	26.50	6.73	1.53	49.07	491.22
XKH	Xingkaihu, Heilongjiang	45°21′54.28″N, 132°19′02.47″E	Lake	5.16	28.7	2.60	0.90	16.03	117.34	1.06	47.68	189.39
HH	Honghe, Heilongjiang	47°34′26.10″N, 133°14′21.24″E	Swamp	4.77	18.54	1.82	0.52	19.64	22.06	1.04	27.10	157.18

### DNA extraction and PCR amplification

Soil DNA was extracted from 0.5 g of fresh sediment sample with a Fast DNA SPIN Kit for Soil (MP Biomedicals, Santa Ana, CA, USA) according to the manufacturer’s instructions. The *phoH* gene was amplified with the degenerate primers vPhoHf (5′-TGC RGG WAC AGG TAA RAC AT-3′) and vPhoHr (5′-TCR CCR CAG AAA AYM ATT TT-3′)^8^. PCR reactions were performed in a 50 μL mixture volume as reported previously (Wang *et al*., 2017). The PCR program parameters included initial denaturation at 95 °C for 5 min, followed by 35 cycles of 95 °C for 1 min, 53 °C for 1 min (annealing), and 72 °C for 1 min (extension), with a final extension at 72 °C for 10 min^8^.

### Cloning and sequencing

The PCR product with a target length of 420 bp was excised from a 2% agarose gel and purified using the QIAExII Gel Extraction Kit (QIAGEN, Shanghai, China). The purified DNA was cloned into the pMD 18-T vector (TaKaRa, Dalian, China) and transformed into competent cells of *Escherichia coli* (*E*. *coli*) DH 5α. White clones were randomly selected, and positive clones were confirmed by PCR amplification. The plasmid DNA of the positive clone was obtained through overnight culture of *E*. *coli* DH 5α and sequenced by a commercial company (BGI, Shenzhen, China). The *phoH* nucleotide sequences obtained in this study were deposited in GenBank under accession numbers MH479451-MH479530 and MH479532-MH479553.

### Sequence analysis

The clone sequences were translated to deduced amino acid sequences using the EMBOSS Transeq program on the European Bioinformatics Institute (EBI) website (https://www.ebi.ac.uk/Tools/st/emboss_transeq/). The closest relatives of the respective *phoH* clones were examined at the amino acid level by the Basic Local Alignment Search Tool (BLAST) search program within the National Center for Biotechnology Information (NCBI) website (https://blast.ncbi.nlm.nih.gov/). References of *phoH* sequences from cultured viruses and bacteria, as well as environmental viral clones, were retrieved from GenBank. The amino acid sequences of *phoH* genes were aligned using ClustalX1.81^32^, and the neighbor-joining phylogenetic tree was constructed by MEGA 6.0 with 1000-fold bootstrap support^33^. The phage *phoH* sequences obtained from the different biomes were defined by the number of operational taxonomic units (OTUs) at sequence divergences of 3%. The NMDS analysis was performed in R-3.4.3 with the ‘vegan’ package. This NMDS analysis employed all published environmental phage *phoH* sequences available at present. The sequence information for this analysis is provided in Supplementary Table S3.

## Supplementary information

Figure S1, Figure S2, Table S1, Table S2, Table S3
