# Supplementary material for: Survey of the bacteriophage phoH gene in wetland sediments in northeast China
Source: Sci Rep. 2019 Jan 29;9:911. doi: 10.1038/s41598-018-37508-4 (PMC6351560; doi:10.1038/s41598-018-37508-4)
Supplement: Supplementary file 1 — Figure S1, Figure S2, Table S1, Table S2, Table S3 [file 41598_2018_37508_MOESM1_ESM.pdf]

Title: Survey of the bacteriophage *phoH* gene in wetland sediments in northeast China

Author names: Xiang Li[1,2], Yan Sun[1,2], Junjie Liu[1], Qin Yao[1] and Guanghua Wang[1,*]

[1]*Key Laboratory of Mollisols Agroecology, Northeast Institute of Geography and Agroecology, Chinese Academy of Sciences, Harbin 150081, China*

[2]*University of Chinese Academy of Sciences, Beijing 100049, China*

[*]**Corresponding author:** Guanghua Wang

Corresponding address: Key Laboratory of Mollisols Agroecology, Northeast Institute of Geography and Agroecology, Chinese Academy of Sciences, Harbin 150081, China

Tel: +86-451-86602745 **Fax:** +86-451-86603736

E-mail address: wanggh@iga.ac.cn

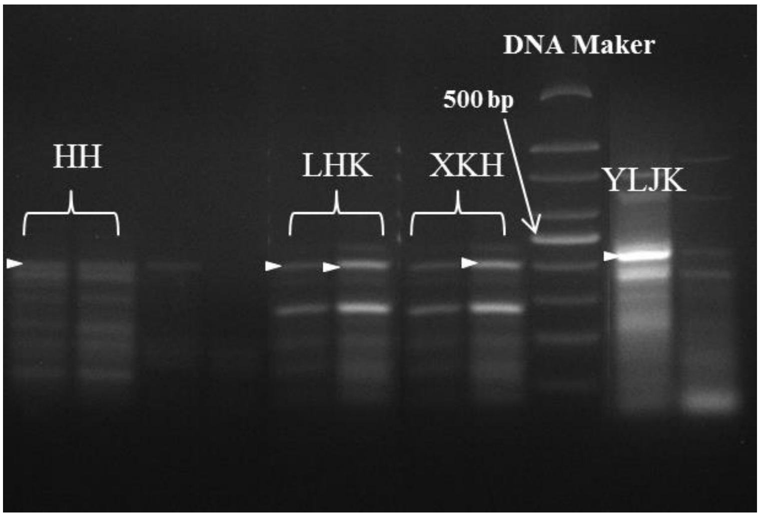

Figure S1. Agarose gel electrophoresis of PCR products generated with the primer set vPhoHf/vPhoHr. Bands of approximately 420 bp marked with white triangles were the potential targeted *phoH* genes that were excised, purified and cloned for sequencing. HH, LHK, XKH and YLJK indicate wetland sediments sampled from Honghe, Liaohekou, Xingkaihu and Yalujiangkou, respectively.

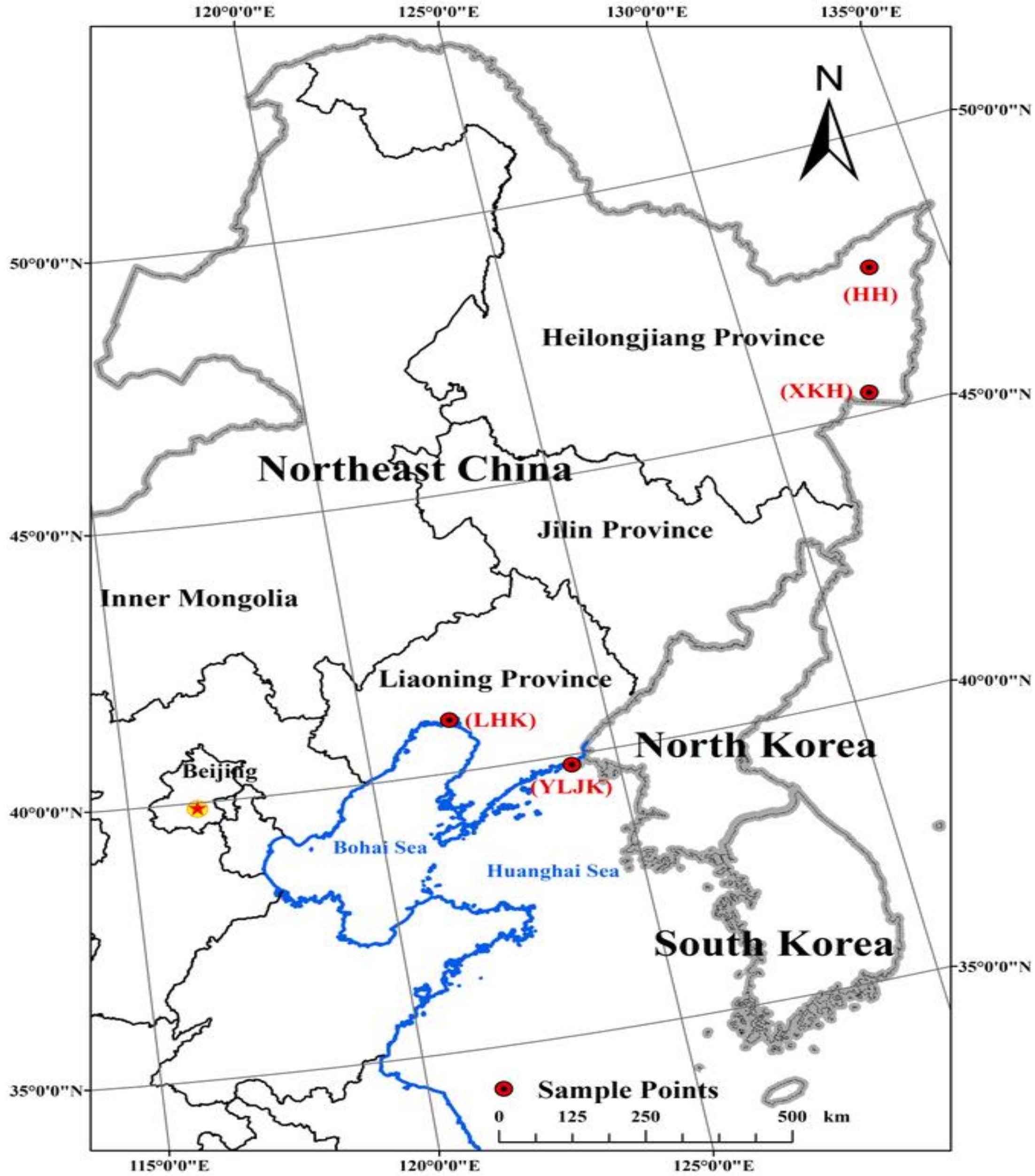

Figure S2. Locations of wetland sampling sites in the map. HH, XKH, LHK and YLJK indicate wetlands of Honghe, Xingkaihu, Liaohekou and Yalujiangkou, respectively.

Table S1. The sequenced DNA *phoH* clones from wetlands had the highest identity to *phoH* sequences of bacteria at the amino acid level.

Clone name[a]	Length[b]	Closest relative			Identity (%)	Alignment	Reference
		Clone/Isolate	Source	Accession number[c]			
YLJK-phoH-42	134	*Draconibacterium orientale*	marine sediment	AHW58931	95	127/133	Li *et al*., 2016
YLJK-phoH-43	134	*Draconibacterium orientale*	marine sediment	AHW58931	95	126/133	Li *et al*., 2016
YLJK-phoH-44	134	*Draconibacterium orientale*	marine sediment	AHW58931	94	125/133	Li *et al*., 2016
YLJK-phoH-45	134	*Draconibacterium orientale*	marine sediment	AHW58931	95	127/133	Li *et al*., 2016
YLJK-phoH-46	134	*Draconibacterium orientale*	marine sediment	AHW58931	95	127/133	Li *et al*., 2016
YLJK-phoH-47	134	*Draconibacterium orientale*	marine sediment	AHW58931	95	126/133	Li *et al*., 2016
YLJK-phoH-48	134	Bacteroidetes bacterium GWE2_32_14	Rifle well CD01	OFX83821	78	104/133	Anantharaman *et al*., 2016
YLJK-phoH-49	134	Ignavibacteria bacterium GWB2_35_6b	Rifle well CD01	OGU35325	86	115/133	Anantharaman *et al*., 2016
YLJK-phoH-50	134	*Draconibacterium orientale*	marine sediment sample	AHW58931	96	128/133	Li *et al*., 2016
YLJK-phoH-51	134	*Draconibacterium orientale*	marine sediment sample	AHW58931	95	127/133	Li *et al*., 2016
YLJK-phoH-52	134	*Draconibacterium orientale*	marine sediment sample	AHW58931	95	127/133	Li *et al*., 2016
YLJK-phoH-53	134	Bacteroidetes bacterium GWE2_32_14	Rifle well CD01	OFX83821	78	104/133	Anantharaman *et al*., 2016
YLJK-phoH-54	134	Bacteroidetes bacterium GWE2_32_14	Rifle well CD01	OFX83821	78	104/133	Anantharaman *et al*., 2016
YLJK-phoH-55	134	*Draconibacterium orientale*	marine sediment sample	AHW58931	94	125/133	Li *et al*., 2016
XKH-phoH-15	134	Spirochaetes bacterium DG_61	Methane-rich estuary sediments 52-54 cm	KPJ86234	79	105/133	Baker *et al*., 2015
XKH-phoH-16	134	*Draconibacterium orientale*	marine sediment sample	AHW58931	85	113/133	Li *et al*., 2016
XKH-phoH-17	134	Bacteroidetes bacterium RIFCSPLOWO2_12_FULL_35_15	Rifle well FP101 under low O_2 conditions; 1.2 micron filter; full assembly	OFY82246	88	117/133	Anantharaman *et al*., 2016
XKH-phoH-18	134	Bacteroidetes bacterium RIFCSPLOWO2_12_FULL_35_15	Rifle well FP101 under low O_2 conditions; 1.2 micron filter; full assembly	OFY82246	86	114/133	Anantharaman *et al*., 2016
XKH-phoH-19	134	Bacteroidetes bacterium RIFCSPLOWO2_12_FULL_35_15	Rifle well FP101 under low O_2 conditions; 1.2 micron filter; full assembly	OFY82246	89	118/133	Anantharaman *et al*., 2016
LHK-phoH-3	134	Bacteroidetes bacterium RIFCSPHIGHO2_02_FULL_44_7	Rifle well FP101 under high O_2 conditions; 0.2 micron filter; full assembly	OFZ12809	89	118/133	Anantharaman *et al*., 2016
LHK-phoH-4	134	Bacteroidetes bacterium RIFCSPHIGHO2_02_FULL_44_7	Rifle well FP101 under high O_2 conditions; 0.2 micron filter; full assembly	OFZ12809	90	120/133	Anantharaman *et al*., 2016
LHK-phoH-5	134	*Draconibacterium orientale*	marine sediment sample	AHW58931	86	114/133	Li *et al*., 2016
LHK-phoH-6	134	*Draconibacterium orientale*	marine sediment sample	AHW58931	86	115/133	Li *et al*., 2016

LHK-phoH-7	131	Spirochaetes bacterium GWB1_48_6	Rifle well CD01 at time point 2/B; 5m depth; 0.1 filter	OHD11197	51	66/130	Anantharaman *et al*., 2016
LHK-phoH-8	134	*Draconibacterium orientale*	marine sediment sample	AHW58931	94	125/133	Li *et al*., 2016
LHK-phoH-9	134	Bacteroidetes bacterium GWE2_32_14	Rifle well CD01	OFX83821	79	105/133	Anantharaman *et al*., 2016
LHK-phoH-10	134	*Draconibacterium orientale*	marine sediment sample	AHW58931	95	126/133	Li *et al*., 2016
LHK-phoH-11	134	*Draconibacterium orientale*	marine sediment sample	AHW58931	86	115/133	Li *et al*., 2016
LHK-phoH-12	134	*Draconibacterium orientale*	marine sediment sample	AHW58931	86	115/133	Li *et al*., 2016
LHK-phoH-13	134	Bacteroidetes bacterium GWE2_32_14	Rifle well CD01	OFX83821	78	104/133	Anantharaman *et al*., 2016
LHK-phoH-14	134	*Draconibacterium orientale*	marine sediment sample	AHW58931	94	125/133	Li *et al*., 2016
LHK-phoH-15	134	*Draconibacterium orientale*	marine sediment sample	AHW58931	84	112/133	Li *et al*., 2016
LHK-phoH-16	134	*Draconibacterium orientale*	marine sediment sample	AHW58931	86	115/133	Li *et al*., 2016
LHK-phoH-17	134	*Draconibacterium orientale*	marine sediment sample	AHW58931	87	116/133	Li *et al*., 2016
LHK-phoH-18	134	*Draconibacterium orientale*	marine sediment sample	AHW58931	86	114/133	Li *et al*., 2016
LHK-phoH-19	134	*Draconibacterium orientale*	marine sediment sample	AHW58931	95	126/133	Li *et al*., 2016
LHK-phoH-20	134	*Draconibacterium orientale*	marine sediment sample	AHW58931	95	126/133	Li *et al*., 2016
LHK-phoH-21	134	*Draconibacterium orientale*	marine sediment sample	AHW58931	95	126/133	Li *et al*., 2016
LHK-phoH-22	134	*Draconibacterium orientale*	marine sediment sample	AHW58931	95	126/133	Li *et al*., 2016
LHK-phoH-23	134	*Draconibacterium orientale*	marine sediment sample	AHW58931	95	126/133	Li *et al*., 2016
LHK-phoH-24	134	*Draconibacterium orientale*	marine sediment sample	AHW58931	94	125/133	Li *et al*., 2016
LHK-phoH-25	134	*Draconibacterium orientale*	marine sediment sample	AHW58931	86	114/133	Li *et al*., 2016
HH-phoH-2	134	Bacteroidetes bacterium RIFCSPLOWO2_12_FULL_35_15	Rifle well FP101 under low O_2 conditions; 1.2 micron filter; full assembly	OFY82246	86	114/133	Anantharaman *et al*., 2016
HH-phoH-3	127	*Methylobacterium extorquens* AM1		ACS38177	51	64/126	Vuilleumier *et al*., 2009

[a]Clone names in YLJK-, XKH-, LHK-, and HH- indicate the clones obtained from wetland sediments of Yalujiangkou, Xinkaihu, Liaohekou, and Honghe, respectively.

[b]The length of amino acid residues.

[c]Accession number of amino acid sequences.

Reference:

Anantharaman, K. *et al*. Thousands of microbial genomes shed light on interconnected biogeochemical processes in an aquifer system. *Nat. Commun*. **7**, 13219 (2016).

Li, X. *et al*. Complete genome sequence of a deeply branched marine Bacteroidia bacterium *Draconibacterium orientale* type strain FH5T. *Mar. Genom.* 26, 13-16 (2016).

Vuilleumier, S. *et al*. Methylobacterium genome sequences: a reference blueprint to investigate microbial metabolism of C1 compounds from natural and industrial sources. PloS one, **4(5)**, e5584 (2009).

Table S2. The sequenced DNA *phoH* clones from wetlands had the highest identity to *phoH* sequences of phage clones at the amino acid level.

Clone name[a]	Length[b]	Closest relative			Identity (%)	Alignment	Reference
		Clone/Isolate	Source	Accession number[c]			
YLJK-phoH-1	135	DA-phoH-18	paddy water	ANW11647	95	127/134	Wang *et al*., 2016
YLJK-phoH-2	135	DA-phoH-18	paddy water	ANW11647	95	127/134	Wang *et al*., 2016
YLJK-phoH-3	135	DA-phoH-18	paddy water	ANW11647	96	128/134	Wang *et al*., 2016
YLJK-phoH-4	135	DA-phoH-26	paddy water	ANW11655	86	115/134	Wang *et al*., 2016
YLJK-phoH-5	135	DA-phoH-26	paddy water	ANW11655	87	116/134	Wang *et al*., 2016
YLJK-phoH-6	130	GOM_phoH_16	Gulf of Mexico	AEQ27534	64	83/129	Goldsmith *et al*., 2011
YLJK-phoH-7	130	GOM_phoH_16	Gulf of Mexico	AEQ27534	64	83/129	Goldsmith *et al*., 2011
YLJK-phoH-8	130	GOM_phoH_16	Gulf of Mexico	AEQ27534	64	83/129	Goldsmith *et al*., 2011
YLJK-phoH-9	130	GOM_phoH_16	Gulf of Mexico	AEQ27534	65	84/129	Goldsmith *et al*., 2011
YLJK-phoH-10	130	GOM_phoH_16	Gulf of Mexico	AEQ27534	64	83/129	Goldsmith *et al*., 2011
YLJK-phoH-11	133	SAR_1000m _16	Sargasso Sea	AEQ27394	48	64/132	Goldsmith *et al*., 2011
YLJK-phoH-12	133	SAR_1000m_16	Sargasso Sea	AEQ27394	48	64/132	Goldsmith *et al*., 2011
YLJK-phoH-13	133	SAR_1000m_16	Sargasso Sea	AEQ27394	48	64/132	Goldsmith *et al*., 2011
YLJK-phoH-14	135	SAR_phoH_25	Sargasso Sea	AEQ27431	87	116/134	Goldsmith *et al*., 2011
YLJK-phoH-15	135	SAR_phoH_25	Sargasso Sea	AEQ27431	87	117/134	Goldsmith *et al*., 2011
YLJK-phoH-16	135	SAR_100m_01	Sargasso Sea	AKV56856	96	128/134	Goldsmith *et al*., 2015
YLJK-phoH-17	135	Phage S-RSM4	Red Sea	YP_003097238	99	132/134	Millard *et al*., 2009
YLJK-phoH-18	135	Phage S-RSM4	Red Sea	YP_003097238	99	132/134	Millard *et al*., 2009
YLJK-phoH-19	135	Phage S-RSM4	Red Sea	YP_003097238	99	132/134	Millard *et al*., 2009
YLJK-phoH-20	135	Phage S-RSM4	Red Sea	YP_003097238	98	131/134	Millard *et al*., 2009
YLJK-phoH-21	135	Phage S-RSM4	Red Sea	YP_003097238	99	132/134	Millard *et al*., 2009
YLJK-phoH-22	134	Phage S-SSM7	Sargasso Sea	YP_004324370	87	116/134	Sullivan *et al*., 2010

YLJK-phoH-23	135	Phage S-SSM7	Sargasso Sea	YP_004324370	87	116/134	Sullivan *et al*., 2010
YLJK-phoH-24	135	Phage S-SSM7	Sargasso Sea	YP_004324370	87	116/134	Sullivan *et al*., 2010
YLJK-phoH-25	135	Phage S-SSM7	Sargasso Sea	YP_004324370	87	116/134	Sullivan *et al*., 2010
YLJK-phoH-26	135	Phage S-SSM7	Sargasso Sea	YP_004324370	87	116/134	Sullivan *et al*., 2010
YLJK-phoH-27	135	Phage S-SSM7	Sargasso Sea	YP_004324370	87	116/134	Sullivan *et al*., 2010
YLJK-phoH-28	135	Phage S-SSM7	Sargasso Sea	YP_004324370	86	115/134	Sullivan *et al*., 2010
YLJK-phoH-29	135	Phage S-SSM7	Sargasso Sea	YP_004324370	87	116/134	Sullivan *et al*., 2010
YLJK-phoH-30	135	Phage S-SSM7	Sargasso Sea	YP_004324370	86	115/134	Sullivan *et al*., 2010
YLJK-phoH-31	135	Phage S-SSM7	Sargasso Sea	YP_004324370	87	116/134	Sullivan *et al*., 2010
YLJK-phoH-32	135	Phage S-SSM7	Sargasso Sea	YP_004324370	87	116/134	Sullivan *et al*., 2010
YLJK-phoH-33	135	Phage S-SSM7	Sargasso Sea	YP_004324370	85	114/134	Sullivan *et al*., 2010
YLJK-phoH-34	135	Phage S-SSM7	Sargasso Sea	YP_004324370	87	116/134	Sullivan *et al*., 2010
YLJK-phoH-35	135	Phage S-SSM7	Sargasso Sea	YP_004324370	86	115/134	Sullivan *et al*., 2010
YLJK-phoH-36	135	Phage S-SSM7	Sargasso Sea	YP_004324370	87	117/134	Sullivan *et al*., 2010
YLJK-phoH-37	135	Phage S-SSM7	Sargasso Sea	YP_004324370	87	117/134	Sullivan *et al*., 2010
YLJK-phoH-38	135	Phage S-SSM7	Sargasso Sea	YP_004324370	87	116/134	Sullivan *et al*., 2010
YLJK-phoH-39	135	Phage S-SSM7	Sargasso Sea	YP_004324370	87	116/134	Sullivan *et al*., 2010
YLJK-phoH-40	135	Phage S-SSM7	Sargasso Sea	YP_004324370	87	116/134	Sullivan *et al*., 2010
YLJK-phoH-41	135	Phage S-WAM2	Padilla Bay	YP_009324389	99	132/134	Crummett *et al*., 2016
LHK-phoH-1	127	YJG-phoH-6	Paddy Water	ANW11801	61	77/126	Wang *et al*., 2016
LHK-phoH-2	135	SAR_100m_01	Sargasso Sea	AKV56856	87	117/134	Goldsmith *et al*., 2015
XKH-phoH-1	127	DA-phoH-166	Paddy Water	ANW11795	96	121/126	Wang *et al*., 2016
XKH-phoH-2	127	DA-phoH-166	Paddy Water	ANW11795	95	120/126	Wang *et al*., 2016
XKH-phoH-3	127	DA-phoH-166	Paddy Water	ANW11795	96	121/126	Wang *et al*., 2016
XKH-phoH-4	129	DA-phoH-29	Paddy Water	ANW11658	90	115/128	Wang *et al*., 2016

XKH-phoH-5	129	SH-phoH-35	Paddy Water	ANW11531	98	125/127	Wang *et al*., 2016
XKH-phoH-6	135	Phage S-SSM7	Sargasso Sea	YP_004324370	87	116/134	Sullivan *et al*., 2010
XKH-phoH-7	126	YJG-phoH-8	Paddy Water	ANW11803	82	102/125	Wang *et al*., 2016
XKH-phoH-8	129	YJG-phoH-90	Paddy Water	ANW11885	99	127/128	Wang *et al*., 2016
XKH-phoH-9	129	YJG-phoH-90	Paddy Water	ANW11885	99	127/128	Wang *et al*., 2016
XKH-phoH-10	129	YJG-phoH-90	Paddy Water	ANW11885	98	126/128	Wang *et al*., 2016
XKH-phoH-11	129	YJG-phoH-90	Paddy Water	ANW11885	99	127/128	Wang *et al*., 2016
XKH-phoH-12	129	YJG-phoH-90	Paddy Water	ANW11885	98	126/128	Wang *et al*., 2016
XKH-phoH-13	129	YJG-phoH-90	Paddy Water	ANW11885	99	127/128	Wang *et al*., 2016
XKH-phoH-14	129	YJG-phoH-90	Paddy Water	ANW11885	100	128/128	Wang *et al*., 2016
HH-phoH-1	129	YJG-phoH-85	Paddy Water	ANW11880	73	94/128	Wang *et al*., 2016
HH-phoH-3	127	*Yersinia* phage phiR1-37	Soil	YP_004934283	53	67/126	Kiljunen *et al*., 2005

[a] Clone names in YLJK-, XKH-, LHK-, and HH- indicate the clones obtained from wetland sediments of Yalujiangkou, Xinkaihu, Liaohekou, and Honghe, respectively.

[b]The length of amino acid residues.

[c] Accession number of amino acid sequences.

References:

Goldsmith, D. B. *et al*. Development of phoH as a novel signature gene for assessing marine phage diversity. *Appl. Environ. Microbiol*. **77**, 7730-7739 (2011).

Goldsmith, D. B., Parsons, R. J., Beyene, D., Salamon, P. & Breitbart, M. Deep sequencing of the viral phoH gene reveals temporal variation, depth-specific composition, and persistent dominance of the same viral phoH genes in the Sargasso Sea. *Peer. J*. **3**, e997 (2015)

Kiljunen, S. *et al*. Yersiniophage phiR1-37 is a tailed bacteriophage having a 270 kb DNA genome with thymidine replaced by deoxyuridine. *Microbiology*, **151**, 4093-4102 (2005).

Sullivan, M. B. *et al*. Genomic analysis of oceanic cyanobacterial myoviruses compared with T4-like myoviruses from diverse hosts and environments. *Environ. Microb*. **12(11)**, 3035-3056 (2010)

Wang, X. Z., Liu, J. J., Yu, Z. H., Jin, J., Liu, X. B. & Wang, G. H. Novel groups and unique distribution of phage *phoH* genes in paddy waters in northeast China. *Sci. Rep*. 6, 38428 (2016).

Table S3. The information of phage *phoH* sequences used for the NMDS analysis

**Source**	**Sample Site**	**NCBI Accession number**	**Number of clones**	**Reference**
Wetland sediment	Yalujiangkou (YLJK)	MH479451 - MH479491	41	This study
	Xingkaihu (XKH)	MH479532-MH479545	14	This study
Paddy water	Daan	KX189635-KX190058	166	Wang *et al*., 2016
	Suihua		97	
	Mudanjiang		36	
	Yanjiagang		125	
Marine water	Raunefjorden	JF963974-JF964153	18	Goldsmith *et al*., 2011
	British Columbia	JF964160-JF964251	18	
	Gulf of Mexico		19	
	Kongsfjorden		19	
	Mediterranean Sea		18	
	Sargasso Sea		69	
	Sargasso Sea 0m		38	
	Sargasso Sea 200m		21	
	Sargasso Sea 500m		24	
	Sargasso Sea 1000m		28	

References:

Wang, X. Z., Liu, J. J., Yu, Z. H., Jin, J., Liu, X. B. & Wang, G. H. Novel groups and unique distribution of phage *phoH* genes in paddy waters in northeast China. *Sci*. *Rep*. **6**, 38428 (2016).

Goldsmith, D. B. *et al*. Development of phoH as a novel signature gene for assessing marine phage diversity. *Appl. Environ. Microbiol*. **77**, 7730-7739 (2011).
